# Supplementary material for: Efficacy and Safety of Bojungikgi-Tang for Persistent Allergic Rhinitis: A Randomized, Double-Blinded, Placebo-Controlled, Phase II Trial
Source: Healthcare (Basel). 2024 May 14;12(10):1017. doi: 10.3390/healthcare12101017 (PMC11120927; doi:10.3390/healthcare12101017)
Supplement: Supplementary file 1 [file healthcare-12-01017-s001.zip › healthcare-2955125-supplementary.pdf]

Table S1. Results for total IgE and eosinophil counts

	**Week 0**	**Week 4**	**Difference**	***P*-value**
Total IgE				
High-dose BJIGT (n=35)	350.92±621.52	363.90±616.03	11.07±122.30	0.8235
Standard-dose BJIGT (n=35)	222.75±391.71	215.96±406.38	-6.79±99.38	0.4703
Placebo (n=35)	256.75±239.64	265.62±232.36	8.87±59.93	-
Eosinophil count				
High-dose BJIGT (n=35)	205.43±184.44	208.53±163.30	0.00±122.45	0.7540
Standard-dose BJIGT (n=35)	145.54±99.51	147.76±82.15	2.22±80.24	0.5308
Placebo (n=35)	256.75±239.64	265.62±232.36	-18.22±145.33	-

Values are expressed as mean±SD in FAS analysis.

IgE, immunoglobulin E; SD, standard deviation; FAS, full analysis set; BJIGT, Bojungikgi-tang

Table S2. Vital signs

		Standard-dose BJIGT (N=35)			High-dose BJIGT (N=35)			Placebo (N=35)			P-Value
BP (systolic)	Baseline	118.26	±	14.14	119.2	±	10.93	123.89	±	10.24	0.116
	Visit 3	118.86	±	13.26	119.34	±	11.25	120.51	±	9.07	0.8205
	Visit 5	118.09	±	10.59	119.24	±	11.95	122.71	±	8.04	0.1662
BP (diastolic)	Baseline	73.2	±	10.09	73.46	±	10.43	74.49	±	7.33	0.4867
	Visit 3	72.34	±	9.32	72.06	±	8.74	72.60	±	9.63	0.9702
	Visit 5	73.46	±	10.43	72.58	±	9.53	75.15	±	9.02	0.4152
Pulse	Baseline	76.63	±	9.16	77.14	±	12.60	79.63	±	12.33	0.5064
	Visit 3	79.69	±	11.24	75.43	±	12.58	76.89	±	9.93	0.2817
	Visit 5	77.27	±	8.44	75.12	±	12.31	77.35	±	8.65	0.5859
Temperature	Baseline	36.39	±	0.22	36.54	±	0.23	36.51	±	0.27	0.0257*
	Visit 3	36.53	±	0.24	36.59	±	0.27	36.46	±	0.21	0.0806
	Visit 5	36.45	±	0.21	36.48	±	0.22	36.5	±	0.27	0.7305`

Values are expressed as mean±SD in FAS analysis. No significant differences were observed among groups except for temperature at baseline, which was within the normal range and had no clinical significance.

Table S3. Laboratory examinations

A: LFT (Liver Function Test)

		Standard-dose BJIGT (N=35)			High-dose BJIGT (N=35)			Placebo (N=35)			P-Value
AST	Baseline	18.66	±	5.17	19.17	±	5.1	19.11	±	6.32	0.9140
	Visit 5	19.97	±	7.82	19.18	±	5.85	19.35	±	4.66	0.8641
ALT	Baseline	18.54	±	11.31	18.43	±	9.98	17.97	±	13.18	0.9764
	Visit 5	18.67	±	13.03	17.76	±	11.55	18.03	±	11.27	0.9511
r-GTP	Baseline	23.51	±	18.11	18.69	±	7.49	28.03	±	28.82	0.1568
	Visit 5	23.76	±	19.23	18.39	±	10.59	26.29	±	27.60	0.2777
ALP	Baseline	61.09	±	20.29	63.63	±	18.89	63.11	±	18.73	0.8443
	Visit 5	58.21	±	20.15	64.42	±	18.26	18.03	±	11.27	0.3359
BUN	Baseline	12.8	±	2.55	12.69	±	3.68	11.48	±	2.53	0.1230
	Visit 5	12.73	±	3.18	11.94	±	2.89	11.61	±	2.7	0.2795
Creatinine	Baseline	0.78	±	0.18	0.81	±	0.17	0.78	±	0.17	0.7473
	Visit 5	0.79	±	0.2	0.79	±	0.17	0.79	±	0.17	0.9967
Total Bilirubin	Baseline	0.62	±	0.28	0.74	±	0.45	0.55	±	0.28	0.0686
	Visit 5	0.61	±	0.3	0.65	±	0.38	0.60	±	0.24	0.7760
Glucose	Baseline	95	±	11.72	95	±	12.19	93.51	±	0.94	0.8108
	Visit 5	96.0	±	7.73	97.36	±	12.88	96.29	±	0.87	0.8387
CPK	Baseline	96.71	±	62.24	136.49	±	140.8	106.43	±	74.78	0.2186
	Visit 5	125.09	±	142.32	156.97	±	222.79	132.71	±	208.92	0.7858
Na	Baseline	139.34	±	1.82	139.71	±	1.93	139.49	±	1.85	0.7067
	Visit 5	138.36	±	0.29	139.03	±	2.13	139.24	±	1.46	0.1563
K	Baseline	4.34	±	0.26	4.43	±	0.34	4.34	±	0.3	0.3468
	Visit 5	4.39	±	0.27	4.34	±	0.33	4.38	±	0.25	0.7156
Cl	Baseline	103.74	±	2.06	103.43	±	1.96	103.37	±	1.78	0.6900
	Visit 5	102.88	±	2.26	103.15	±	2.27	103.56	±	1.69	0.4087
PT-INR	Baseline	1.02	±	0.16	1.95	±	2.95	1.04	±	0.08	0.0381*
	Visit 5	2.52	±	8.47	1.35	±	1.73	1.05	±	0.07	0.4461
aPTT	Baseline	30.11	±	2.59	29.77	±	2.20	29.0	±	2.28	0.136
	Visit 5	30.95	±	4.42	29.5	±	2.58	29.6	±	3.13	0.1672

B. CBC (Complete Blood Counts)

		Standard-dose BJIGT (N=35)			High-dose BJIGT (N=35)			Placebo (N=35)			P-Value
RBC	Baseline	4.76	±	0.42	4.77	±	0.35	4.76	±	0.47	0.9970
	Visit 5	4.71	±	0.48	4.72	±	0.35	4.75	±	0.45	0.9145
Hemoglobin	Baseline	14.0	±	1.34	13.85	±	1.4	13.93	±	1.61	0.9160
	Visit 5	13.91	±	1.53	17.52	±	21.5	14.84	±	5.64	0.4946
Hematocrit	Baseline	41.91	±	4.0	41.85	±	3.61	42.10	±	4.48	0.9655
	Visit 5	41.84	±	4.38	41.34	±	3.96	42.16	±	4.51	0.7331
WBC	Baseline	5.46	±	1.29	5.05	±	1.26	5.89	±	1.32	0.0289*
	Visit 5	5.69	±	1.28	5.16	±	1.47	5.75	±	1.35	0.1559
Netrophil	Baseline	58.87	±	6.74	58.79	±	7.57	60.38	±	7.24	0.5833
	Visit 5	60.67	±	13.1	59.25	±	8.5	58.57	±	7.48	0.6810
Monocyte	Baseline	7.08	±	1.61	29.01	±	123.55	7.41	±	2.38	0.3416
	Visit 5	7.99	±	5.68	7.56	±	2.07	7.13	±	1.63	0.6208
Lymphocyte	Baseline	30.67	±	6.21	29.27	±	6.78	28.98	±	6.77	0.5193
	Visit 5	27.06	±	8.68	29.15	±	7.52	30.63	±	7.55	0.1855
Eosinophil	Baseline	2.98	±	2.0	3.51	±	2.57	2.84	±	1.24	0.3401
	Visit 5	2.37	±	1.26	3.68	±	2.52	3.21	±	1.82	0.0227*
Basophil	Baseline	0.41	±	0.18	0.62	±	0.91	0.39	±	0.21	0.1659
	Visit 5	0.4	±	0.21	0.43	±	0.48	0.46	±	0.19	0.7111
Platelet	Baseline	27.61	±	6.2	25.83	±	5.89	26.89	±	5.4	0.4416
	Visit 5	27.32	±	5.61	25.82	±	6.41	27.44	±	4.3	0.4098
ESR	Baseline	10.46	±	7.07	10.77	±	6.55	9.91	±	9.71	0.9000
	Visit 5	9.88	±	7.3	10.61	±	6.88	9.82	±	8.87	0.8998

C. U/A (Urinalysis)

		High-dose BJIGT (N=35)			Placebo (N=35)			Standard-dose BJIGT (N=35)			P-Value
SG	Baseline	1.02	±	0.01	1.02	±	0.0	1.03	±	0.05	0.5472
	Visit 5	1.32	±	1.23	1.02	±	0.0	1.14	±	0.68	0.3218
PH	Baseline	5.70	±	0.87	5.74	±	0.91	5.91	±	0.84	0.5572
	Visit 5	5.58	±	1.28	5.71	±	0.78	5.82	±	0.79	0.6287

Values are expressed as mean±SD in FAS analysis. No significant differences were observed among groups. In some cases where differences were observed, they were within the normal range and lacked clinical significance.
